# Phase II randomised discontinuation trial of the MET/VEGF receptor inhibitor cabozantinib in metastatic melanoma

**DOI:** 10.1038/bjc.2016.419

**Published:** 2017-01-19

**Authors:** Adil Daud, Harriet M Kluger, Razelle Kurzrock, Frauke Schimmoller, Aaron L Weitzman, Thomas A Samuel, Ali H Moussa, Michael S Gordon, Geoffrey I Shapiro

**Affiliations:** 1 University of California, San Francisco Medical Center at Parnassus, 1600 Divisadero Street, MZ Bldg A, San Francisco, CA 94115, USA; 2 Yale Cancer Center, Yale University School of Medicine, 333 Cedar Street, PO Box 208028, New Haven, CT 06520-8028, USA; 3 University of California, San Diego Moores Cancer Center, 3855 Health Sciences Drive, MC #0658, La Jolla, CA 92093-0658, USA; 4 Exelixis Inc., 210 E. Grand Avenue, South San Francisco, CA 94080, USA; 5 Department of Hematology/Oncology, Cleveland Clinic Florida, 2950 Cleveland Clinic Blvd, Weston, FL 33331, USA; 6 Cancer Care Associates, 1810 E 15th Street, Tulsa, OK 74104, USA; 7 Pinnacle Oncology Hematology, 9055 E. Del Camino, Suite 100, Scottsdale, AZ 85258, USA; 8 Department of Medical Oncology, Dana-Farber Cancer Institute, Early Drug Development Center, 450 Brookline Avenue, Boston, MA 02215, USA

**Keywords:** cabozantinib, metastatic melanoma, uveal melanoma, vascular endothelial growth factor receptor, MET/VEGFR inhibitor, bone metastases

## Abstract

**Background:**

A phase II randomised discontinuation trial assessed cabozantinib (XL184), an orally bioavailable inhibitor of tyrosine kinases including VEGF receptors, MET, and AXL, in a cohort of patients with metastatic melanoma.

**Methods:**

Patients received cabozantinib 100 mg daily during a 12-week lead-in. Patients with stable disease (SD) per Response Evaluation Criteria in Solid Tumours (RECIST) at week 12 were randomised to cabozantinib or placebo. Primary endpoints were objective response rate (ORR) at week 12 and postrandomisation progression-free survival (PFS).

**Results:**

Seventy-seven patients were enroled (62% cutaneous, 30% uveal, and 8% mucosal). At week 12, the ORR was 5% 39% of patients had SD. During the lead-in phase, reduction in target lesions from baseline was seen in 55% of evaluable patients overall and in 59% of evaluable patients with uveal melanoma. Median PFS after randomisation was 4.1 months with cabozantinib and 2.8 months with placebo (hazard ratio of 0.59; *P*=0.284). Median PFS from study day 1 was 3.8 months, 6-month PFS was 33%, and median overall survival was 9.4 months. The most common grade 3/4 adverse events were fatigue (14%), hypertension (10%), and abdominal pain (8%). One treatment-related death was reported from peritonitis due to diverticular perforation.

**Conclusions:**

Cabozantinib has clinical activity in patients with metastatic melanoma, including uveal melanoma. Further clinical investigation is warranted.

Historically, metastatic melanoma has been a challenging disease to manage, with traditional chemotherapy having no effect on the median survival time of 6–10 months (Tsao *et al*, 2004). A number of new therapies for the treatment of stage 4 melanoma have recently become available, including the immuno-oncology agents ipilimumab, nivolumab, and pembrolizumab (Hodi *et al*, 2010; Robert *et al*, 2014, 2015), as well as inhibitors of protein kinases BRAF and mitogen-activated protein kinase (MAPK)/ERK kinase (MEK) for use in patients with activating mutations in *BRAF* (Hauschild *et al*, 2012; Sosman *et al*, 2012; Larkin *et al*, 2014; Long *et al*, 2014). Although these new agents have extended median progression-free survival (PFS) and overall survival (OS) in the treated populations, and induced durable responses, the majority of patients will eventually develop progressive melanoma and require additional therapy. In addition, the uveal melanoma subtype usually lacks *BRAF* mutations (Edmunds *et al*, 2003; Rimoldi *et al*, 2003), and with the exception of selumetinib, few data exist supporting the use of BRAF or MEK inhibitors in uveal melanoma (Carvajal *et al*, 2014). Pembrolizumab and ipilimumab have also shown signs of clinical activity in uveal melanoma in early phase trials (Kottschade *et al*, 2016; Oliva *et al*, 2016).

The receptor tyrosine kinase MET and its cognate ligand hepatocyte growth factor (HGF) have been implicated in diverse aspects of tumour pathobiology, including tumour growth, survival, neoangiogenesis, invasion, and dissemination (Gherardi *et al*, 2012). MET pathway activation and dysregulation have been implicated in multiple cancers, including melanoma. In a survey of 40 malignant melanoma specimens, MET expression and activation were evident in 88% and 21% of cases, respectively (Puri *et al*, 2007). In a genomic survey, the gene encoding MET was amplified and overexpressed in metastatic melanomas compared with primary melanomas (Kabbarah *et al*, 2010). In addition to the direct role of MET signalling in melanoma, HGF expression by stromal cells has been linked to innate resistance to RAF inhibitor treatment in melanoma patients. Moreover, amplification of the gene encoding MET has been implicated in acquired resistance to the BRAF inhibitor vemurafenib in cultured melanoma cells (Vergani *et al*, 2011; Straussman *et al*, 2012).

Uveal melanoma is a particularly treatment-resistant melanoma subtype that is often excluded from clinical trials because of perceived poor response rates, low survival, and a high incidence of liver metastases. However, MET may be a rational target for the uveal subtype. Mutations in the *GNAQ* and *GNA11* genes, which encode guanine nucleotide-binding protein alpha subunits, are found in up to 83% of uveal melanomas (Van Raamsdonk *et al*, 2010). These mutations can lead to upregulation of MET, which is implicated in proliferation and migration of uveal melanoma cells (Patel *et al*, 2011; Yeh *et al*, 2011). In particular, preclinical evidence suggests that liver metastasis from uveal melanoma may be strongly dependent on the MET pathway (Patel *et al*, 2011; Yeh *et al*, 2011).

The receptor tyrosine kinase AXL is expressed in cutaneous and uveal primary melanomas and cell lines and regulates cell growth, survival, and migration (van Ginkel *et al*, 2004; Sensi *et al*, 2011). AXL expression negatively correlates with expression of microphthalmia-associated transcription factor (MITF), and a high AXL/MITF ratio is associated with early resistance to BRAF inhibitors (Sensi *et al*, 2011; Muller *et al*, 2014).

Another protein that has a key role in melanoma is VEGF, as it is a central mediator of tumour angiogenesis and lymphangiogenesis (Carmeliet and Jain, 2011) and is dysregulated in melanoma. Circulating levels of VEGF, VEGFR1, and VEGFR3 are elevated in melanoma patients, and have been linked to poor prognosis (Tas *et al*, 2006; Mouawad *et al*, 2009).

Multiple agents that target VEGF signalling have been explored as monotherapy in melanoma, including bevacizumab, aflibercept, axitinib, vatalanib, sunitinib, dovitinib, and sorafenib; however, only a few demonstrated modest clinical activity (Nikolaou *et al*, 2012). The development of resistance to targeted monotherapy can limit clinical efficacy. A variety of preclinical models and clinical experience suggest that selective inhibition of VEGFR signalling may lead to a resistant phenotype (Ebos *et al*, 2009; Loges *et al*, 2009; Aftab and McDonald, 2011; Sennino *et al*, 2012) and imply that a multitargeted approach may be more effective. Indeed, combined inhibition of VEGFR and MET pathways resulted in enhanced efficacy over inhibition of either pathway alone in a mouse neuroendocrine tumour model (Sennino *et al*, 2012).

Cabozantinib (XL184; Exelixis, Inc.) is an orally bioavailable tyrosine kinase inhibitor that targets multiple receptor tyrosine kinases including MET, AXL, and VEGFRs. Cabozantinib potently inhibits HGF-induced migration and invasion of B16F10 melanoma cells (Yakes *et al*, 2011). Moreover, cabozantinib has shown activity in a xenograft model of uveal melanoma metastatic to the liver (Yeh *et al*, 2011). In a phase I clinical study, treatment with cabozantinib resulted in tumour reduction in multiple cancer types (Kurzrock *et al*, 2011).

The randomised discontinuation trial is used to determine the clinical activity of a therapeutic agent and minimise the use of placebo (Amery and Dony, 1975; Kopec *et al*, 1993). During the open-label phase, all patients receive the study medication for a predetermined time-period. Patients who achieve a tumour response continue open-label treatment, whereas those who achieve stable disease (SD) during the open-label phase are randomised to either continue treatment or receive placebo during the double-blind phase. The randomised discontinuation trial design enriches for a potentially sensitive population and then examines whether achieving SD was due to the therapeutic agent or selection of an indolent disease group (Stadler, 2009).

The current phase II placebo-controlled, randomised discontinuation trial of cabozantinib was conducted in nine selected tumour types, including castration-resistant prostate cancer, hepatocellular carcinoma, non-small-cell lung cancer, ovarian cancer, melanoma, and breast cancer (ClinicalTrials.gov: NCT00940225; Gordon *et al*, 2011). This report describes the results of this trial in the cohort of patients with melanoma, including cutaneous, uveal, and mucosal subtypes.

## Materials and methods

### Patients

Eligible patients had histologically confirmed melanoma (including cutaneous, uveal, and mucosal subtypes) with measurable disease by Response Evaluation Criteria in Solid Tumours (RECIST) version 1.0 (Therasse *et al*, 2000) and progressive disease at screening. Other eligibility requirements have been previously described and included a requirement for Eastern Cooperative Oncology Group (ECOG) performance status of 0 or 1 (Smith *et al*, 2013). Patients had no more than two prior standard or investigational chemotherapy or targeted therapy regimens in the metastic setting completed ⩾4 weeks before study entry. Radiotherapy and immunotherapy (such as IL-2 and immune checkpoint inhibitors) did not count towards this restriction. Patients with known brain metastases, radiation therapy within 2 weeks, or clinically significant intercurrent illness were excluded. The study was conducted in accordance with the Declaration of Helsinki and Good Clinical Practice guidelines. The study protocol and informed consent documents were reviewed and approved by the Institutional Review Boards of the participating institutions, and informed consent was obtained from all patients before any study-specified procedures.

### Study design

The primary endpoint of the lead-in phase was objective response rate at week 12, and the primary endpoint of the randomised phase was PFS. Secondary endpoints included assessing the safety and tolerability of the agent and potential pharmacodynamic effects. The study was designed as a randomised discontinuation trial (Ratain *et al*, 2006), and all patients received open-label cabozantinib treatment during a 12-week lead-in stage (Supplementary Figure S1). At week 12, patients with evidence of response by RECIST (⩾30% decrease in the sum of measurable lesions) remained on open-label cabozantinib, and patients with progressive disease (Therasse *et al*, 2000) were discontinued. Patients who did not meet the criteria for response or progression were judged to have SD and were randomised to either placebo or cabozantinib in double-blinded fashion. All randomised patients were followed until progression, at which point treatment assignment was unblinded; patients who were receiving cabozantinib were discontinued, and those receiving placebo were offered the opportunity to restart cabozantinib. Patients who chose to cross over to cabozantinib after progression on placebo were followed until their subsequent progression on open-label cabozantinib. The protocol was amended to add follow-up for OS. A study oversight committee monitored efficacy during the lead-in stage and had the ability to suspend randomisation based on the reviewed data. An independent data-monitoring committee monitored safety in the blinded randomised stage.

### Study drug administration

Patients received cabozantinib at a daily oral dose of 100 mg (free base equivalent weight) during a 12-week open-label lead-in stage. Details of dosing interruptions have been previously described (Smith *et al*, 2013). If treatment was held to manage an adverse event (AE) related to treatment, cabozantinib was subsequently resumed at a reduced dose. Interruption in dosing for up to 3 weeks was allowed.

### Study assessments

Safety parameters were evaluated every 3 weeks, and tumours were assessed every 6 weeks throughout the study. Efficacy assessments included radiographic soft tissue (by computed tomography (CT) or magnetic resonance imaging) and bone imaging by bone scan for patients with a history of bone metastasis. The PFS analysis was conducted based on investigator-assessed response by RECIST 1.0. Bone scan changes were assessed by an independent radiology facility (MedQIA; Los Angeles, CA, USA).

Other clinical assessments included medical and cancer history, physical examination, vital signs and body weight, electrocardiography, ECOG performance status, safety laboratories (serum chemistry, haematology, coagulation, and urinalysis), concomitant medications, AEs, and information on subsequent anticancer treatment. Other exploratory endpoints included analysis of changes in the circulating bone biomarker C-terminal cross-linked telopeptide of type I collagen (CTx), assessment of bone scan resolution (when applicable), and analysis of *BRAF* and/or *GNAQ/GNA11* mutational status of tumour samples. The data cutoff date for the results presented in this publication was 16 December 2011.

### Statistical considerations

The study employed an adaptive design. An SD rate of 35% in a cohort during the lead-in stage was selected as a reasonable response rate that indicated sufficient preliminary efficacy to evaluate the cohort further. Up to 200 patients could be enroled to a tumour type cohort to randomise 70 patients and achieve 52 events postrandomisation. This design provided 80% power to detect a hazard ratio of 0.5 for PFS after randomisation. Enrolment into a cohort could be halted if an insufficient number of patients had disease stabilisation due to either high or low rates of clinical activity during the lead-in stage. For the analysis of PFS from date of randomisation and OS from first dose, the Kaplan–Meier method was employed to estimate medians, and the log-rank test was used for inference testing. The Cox proportional hazards model was used to estimate hazard ratio. For the analysis of overall PFS from first dose, including the lead-in stage, the estimation method as described by Ratain *et al* (Ratain *et al*, 2006) was utilised. All treated patients contributed to the PFS estimate through the first 12 weeks. After week 12, the PFS was estimated as a weighted average of those continuing on open-label treatment and those randomised to cabozantinib. The weights corresponded to the fraction of patients continuing on open-label treatment at week 12 and the proportion of patients randomised at week 12 (including placebo).

## Results

### Patients

**Figure 1:**
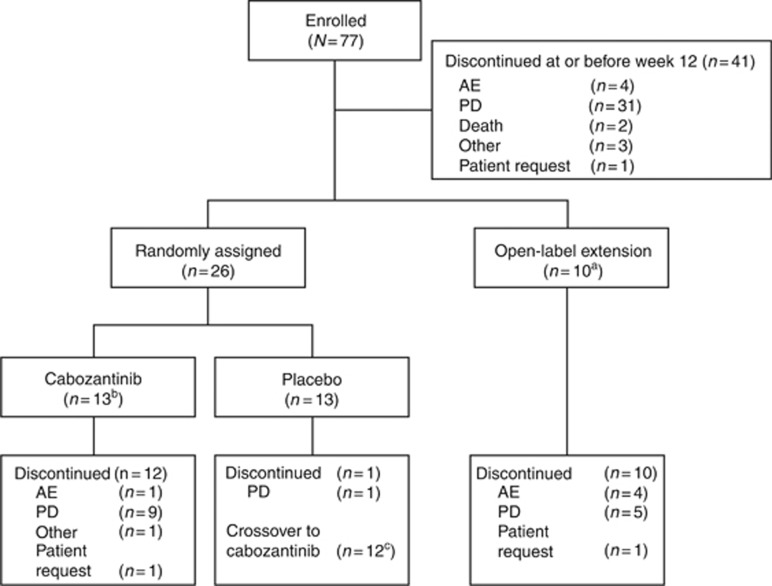
**CONSORT diagram.**^a^Patients who were neither randomised nor had discontinued treatment during the lead-in stage are shown in the open-label continuation group, including five patients with SD at week 12 based on final data analysis. Randomisation was halted early by recommendation of the study oversight committee due to the high rates of tumour regression and the observation of symptomatic progression in individual patients randomised to placebo in several of the disease cohorts. ^b^One patient originally randomised to cabozantinib remained active at the time of the data cutoff. ^c^Twelve patients were randomly assigned to placebo and crossed over to open-label cabozantinib after unblinding. Four of those 12 patients remained active at the time of the data cutoff, and eight discontinued treatment (one patient request, one death, and six PD). CONSORT=Consolidated Standards of Reporting Trials; PD=progressive disease.

From September 2009 to November 2010, 77 patients with metastatic melanoma were enroled in the United States, Belgium, and Israel. Baseline demographic and clinical characteristics are summarised in Table 1. Thirty per cent of patients had uveal melanoma, and 22% had bone metastases. Among the 54 patients with available mutation data, *BRAF* mutations were detected in 31%. Sixty-six per cent of patients had at least one line of prior systemic therapy. During the 12-week lead-in stage, 41 of the 77 enroled patients (53%) discontinued study treatment primarily because of progressive disease (Figure 1). At week 12, 26 patients (34%) were randomised to receive either cabozantinib or placebo, and 10 patients (13%) continued open-label treatment with cabozantinib (Figure 1). Five of these 10 patients had experienced a partial response (PR); 4 of these were later determined to be confirmed PRs. The remaining five patients had SD at week 12 based on the final data analysis (Figure 1). Although the goal of the study was to randomise ∼70 patients per cohort at week 12, randomisation was halted early by recommendation of the study oversight committee due to the high rates of tumour regression and the observation of symptomatic progression in individual patients randomised to placebo in several of the disease cohorts (Smith *et al*, 2013). Mean cabozantinib plasma concentration in patients who received at least 14 of 15 uninterrupted 100 mg per day cabozantinib doses over the 2 weeks before the end of the week 6 pharmacokinetics sampling visit (*n*=33) was 1118 ng ml^−1^ (±542 ng ml^−1^), with a corresponding per cent coefficient of variation of 48.5%.

### Response

**Figure 2:**
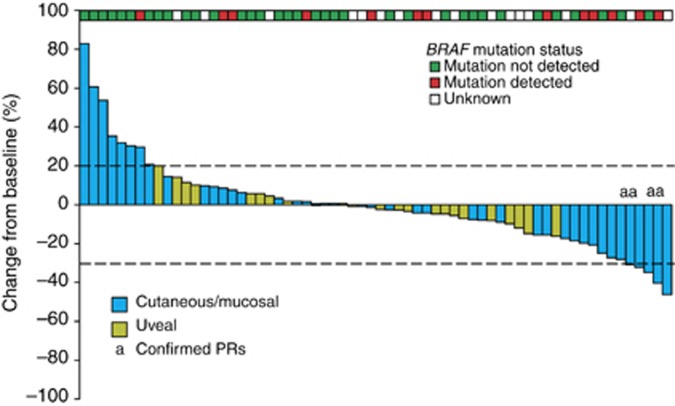
**Best change from baseline in investigator-assessed measurements of target lesions using RECIST (version 1.0) was determined for patients who had baseline and at least one evaluable postbaseline radiographic scan in the first 12 weeks (*n*=64).***BRAF* mutation status is based on sponsor analyses of archival tumour tissue and investigator reporting. RECIST=Response Evaluation Criteria in Solid Tumours.

The primary endpoint for the open-label, 12-week lead-in stage of the study was response rate per RECIST 1.0. Among the 77 patients enroled in the lead-in stage, the objective response rate at week 12 was 5%, with 4 patients having a confirmed PR. An additional 30 patients had SD at week 12, resulting in a disease control rate (PR+SD) of 44%. Eleven patients were not evaluable for response per RECIST due to the lack of adequate postbaseline scan data. Of 64 patients evaluable for change in measurable disease, 35 (55%) had at least one assessment showing a reduction of measurable target lesions, including 13 of the 22 evaluable patients (59%) with uveal melanoma (Figure 2). Reduction in measurable disease appeared to be independent of *BRAF* mutation status.

### Progression-free survival and overall survival

**Figure 3:**
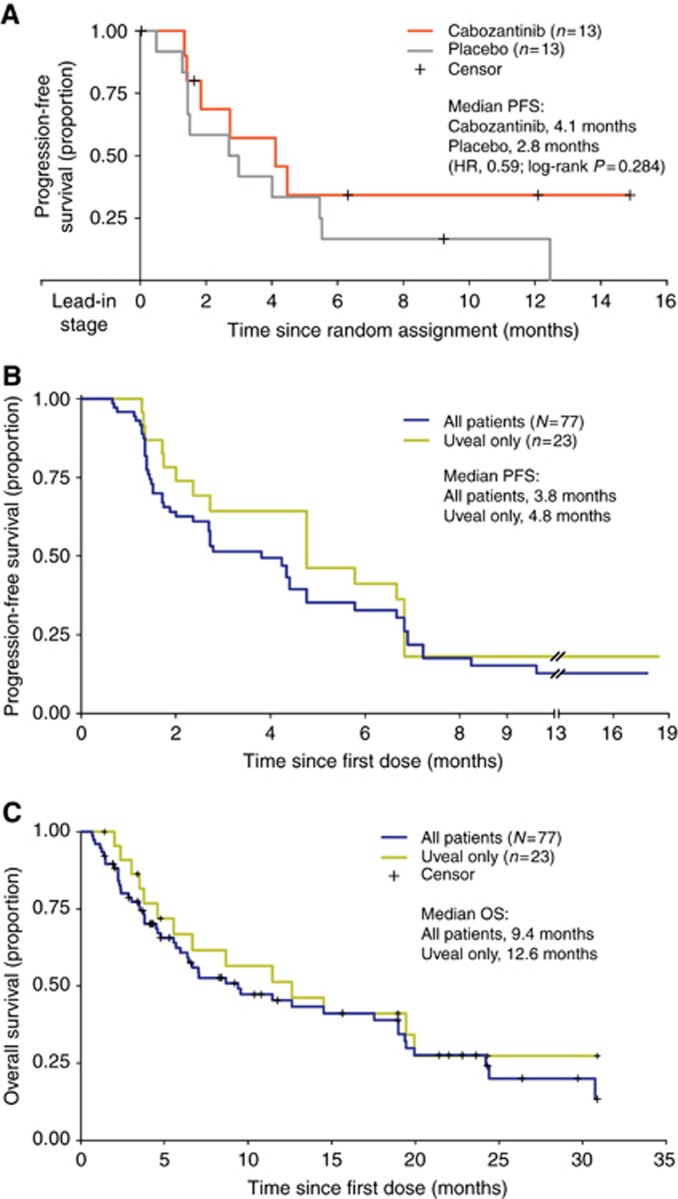
**Estimates of PFS and OS.**(**A**) PFS from week 12 in patients with melanoma who were randomly assigned to continue treatment with cabozantinib (*n*=13) or placebo (*n*=13). (**B**) PFS from first dose in all patients with metastatic melanoma (*N*=77) and in patients with uveal melanoma (*n*=23). (**C**) OS in all patients with metastatic melanoma (*N*=77) and in patients with uveal melanoma (*n*=23). HR=hazard ratio.

The primary endpoint for the randomised stage of the study was PFS after week 12 for patients with SD who were randomised to blinded treatment with cabozantinib or placebo. Twenty-six patients with SD at week 12 were randomised to cabozantinib (*n*=13) or placebo (*n*=13). The median PFS after randomisation at week 12 for cabozantinib patients was 4.1 months (95% confidence interval=1.8 months, not reached), and 2.8 months for placebo patients (95% confidence interval=1.5-5.5 months), with a hazard ratio of 0.59 (*P*=0.284; Figure 3A). Following documented disease progression, 12 patients in the placebo group elected to cross over to open-label cabozantinib therapy (Figure 1).

The median overall PFS for all treated patients (*N*=77) over the entire treatment period from the start of therapy (week 1 day 1) was 3.8 months, and the PFS rate at month 6 was 33% (Figure 3B). Median OS was 9.4 months for all treated patients (Figure 3C).

### Uveal melanoma cohort

The melanoma cohort included 23 patients with metastatic uveal melanoma. The median age was 65, and the median number of prior systemic anticancer regimens was one. Patients had substantial tumour burden at baseline, as reflected by a median sum of the longest diameter (SLD) of target lesions of 11.9 cm (range, 2.0–37.2), and hepatic metastases were present in 16 patients (70%). The majority (9/10) of patient samples analysed for *GNAQ/GNA11* mutation status harboured either a *GNAQ* (*n*=5) or *GNA11* mutation (*n*=4) (Table 2). *GNA11* status was unknown in the tissue sample from one patient with no detectable *GNAQ* mutation.

In the uveal melanoma cohort, 61% of patients (14/23) had SD at week 12, and no patient had a PR, resulting in an overall disease control rate of 61%. The median PFS for the 23 patients with uveal melanoma was 4.8 months (41% PFS rate at 6 months; Figure 3B), and median OS was 12.6 months (Figure 3C). Most patients with uveal melanoma stayed on study treatment for >4 months, and six patients stayed on treatment for >10 months (Table 2). Two patients with uveal melanoma had bone metastases and a baseline bone scan, and both experienced partial resolution of their bone lesions during treatment with cabozantinib (Supplementary Figure S2). Both patients also experienced pain relief (per investigator) and had prolonged clinical benefit of ∼12 and 6 months.

Because of the frequent occurrence and correlative morbidity and mortality of hepatic metastases observed in patients with uveal melanoma (Spagnolo *et al*, 2012), an additional exploratory analysis of best change in the SLDs of hepatic lesions through week 12 was performed for the 16 patients in the uveal cohort who had hepatic metastases at baseline. Among those patients, six experienced a decrease of any magnitude (range, 5–21% decrease) in the SLD, two experienced no change, and eight experienced an increase of any magnitude (range, 2–28% increase). Five of the six patients with a decrease in SLD received treatment beyond the lead-in stage. All five of these patients were randomised to placebo at week 12, and in the absence of cabozantinib treatment, each of these patients experienced hepatic lesion growth at the subsequent assessment at week 18.

### Bone marker analysis

The bone resorption marker CTx is often elevated in patients with bone metastases and provides an objective measure of the effect of therapy on the rate of bone turnover. CTx levels were analysed in plasma samples from 13 patients with bone metastases. Eleven of these 13 patients had reductions in CTx ranging from 17 to 93%, and seven of these 13 patients (54%) had reductions of >50% (Supplementary Figure S3). Two of these 13 patients were treated with bisphosphonate, and for both the reduction in CTx was >80%.

### Safety

The most frequently reported AEs during the lead-in stage of the study, regardless of causality, are listed in Table 3. The most common (⩾5%) grade 3/4 events were fatigue (14%), hypertension (10%), abdominal pain (8%), hand-foot syndrome (5%), asthenia (5%), back pain (5%), and hypokalaemia (5%). During the 12-week lead-in stage, six patients (8%) discontinued study treatment because of AEs. Two of these (3%) were due to a grade 5 event: one patient died from peritonitis due to diverticular perforation (deemed related), and one patient died from an unknown cause (deemed unrelated). Twenty-two patients (29%) had at least one dose reduction during the lead-in stage.

## Discussion

There have been remarkable advances recently in the treatment of patients with metastatic melanoma, including the development of inhibitors to immune checkpoint proteins and targeted therapies against the protein kinases BRAF and MEK. Ipilimumab, an anti-CTLA4 antibody, and nivolumab and pembrolizumab, antibodies directed against PD-1, have demonstrated significant clinical activity in advanced melanoma including improvements in PFS and OS and induction of durable responses (Hodi *et al*, 2010; Robert *et al*, 2014; Larkin *et al*, 2015; Robert *et al*, 2015).

In a pivotal phase III randomised trial in patients with *BRAF*-mutant melanoma, vemurafenib achieved an objective response rate of 48% and median PFS of 5.3 months versus 5% and 1.6 months with dacarbazine, respectively (Chapman *et al*, 2011; Kudchadkar *et al*, 2013). Median OS was 13.2 months for vemurafenib versus 9.6 months for dacarbazine (Kudchadkar *et al*, 2013). Significantly improved PFS has been observed in *BRAF*-mutant melanomas when BRAF and MEK inhibitors are used in combination (Larkin *et al*, 2014; Long *et al*, 2014); median PFS of 9.9 months was reported from a study of vemurafenib+cobimetinib, and PFS of 9.3 months was reported from a study of dabrafenib+trametinib. On extended follow-up, median OS for vemurafenib+cobimetinib was 22.3 months versus 17.4 months for vemurafenib alone, and median OS for dabrafenib+trametinib for 25.1 versus 18.7 months for dabrafenib alone (Flaherty *et al*, 2016; McArthur *et al*, 2016). However, the development of treatment resistance typically occurs after exposure to BRAF and MEK inhibitors, and there remains a need for additional treatment options. Also, it should be noted that only ∼50% of melanomas carry the *BRAF* mutation (Davies *et al*, 2002), and that *BRAF* mutations are very rare in uveal melanoma (Edmunds *et al*, 2003; Rimoldi *et al*, 2003).

There is a strong rationale for targeting MET, AXL, and VEGFRs in melanoma. MET activation, which may be driven by stromal HGF expression, has been shown to mediate melanoma growth, dissemination, and resistance to BRAF inhibition in multiple preclinical models (Puri *et al*, 2007; Kabbarah *et al*, 2010; Vergani *et al*, 2011; Straussman *et al*, 2012). AXL also regulates melanoma growth and migration and has been implicated in resistance to BRAF inhibition (van Ginkel *et al*, 2004; Sensi *et al*, 2011; Muller *et al*, 2014). VEGFR kinase inhibitors and anti-VEGF antibodies are part of standard therapy for numerous solid tumour malignancies and have been explored in melanoma (Nikolaou *et al*, 2012). Although activity has been observed with axitinib, pazopanib, and bevacizumab (Nikolaou *et al*, 2012), none of these agents have been approved in melanoma. Preclinical data indicate that a combination of VEGF pathway and MET inhibition can be effective in targeting tumour angiogenesis and suppressing tumour growth and metastasis (Shojaei *et al*, 2010; Aftab and McDonald, 2011; Sennino *et al*, 2012; Shojaei *et al*, 2012). We therefore included a cohort of metastatic melanoma patients in a randomised discontinuation trial designed to explore the activity of cabozantinib in nine solid tumour types.

The results of the current analysis demonstrate single-agent activity of cabozantinib in patients with uveal, cutaneous, or mucosal melanoma. At 12 weeks following the lead-in stage, the objective response rate by RECIST was modest (5%); however, 57% of patients experienced SD. Furthermore, the median OS (9.4 months), PFS (3.8 months), and PFS rate at 6 months (33%) are noteworthy in this setting.

The current study was underpowered to detect a difference in PFS between cabozantinib and placebo because the number of patients randomised at 12 weeks did not achieve the protocol-specified number. Randomisation was halted early by recommendation of the study oversight committee due to the high rates of tumour regression and the observation of symptomatic progression in individual patients randomised to placebo in several of the disease cohorts.

Twenty-six melanoma patients with SD after 12 weeks of cabozantinib treatment were randomised to cabozantinib or placebo. Among these patients, cabozantinib treatment resulted in a median PFS of 4.1 months versus 2.8 months for placebo. The difference in PFS was not statistically significant, possibly due to the small number of randomised patients.

*BRAF* mutation status was determined in available tumour tissue from 54 of the 77 enroled patients. There was no apparent association between *BRAF* mutation status and clinical outcome, and tumour reduction was observed in patients both with and without detectable *BRAF* mutations in their tumours. Interestingly, MET activation has been identified as a mechanism of resistance to BRAF inhibition in melanoma (Vergani *et al*, 2011). Therefore, it is reasonable to surmise that combining a BRAF inhibitor and cabozantinib may be a useful approach in patients with *BRAF* mutation-positive tumours and may delay or prevent the development of resistance. However, future studies are needed to investigate this possibility.

Although the patient population was not deliberately enriched for uveal melanoma, this subtype represents a relatively high fraction of patients (30%) given its low incidence, presumably due to the lack of effective treatment options. Uveal melanoma is typically associated with poor response rates, a high incidence of liver metastases (up to 90% of patients with metastatic disease), and a short median survival (3–5 months) (Patel *et al*, 2011; Spagnolo *et al*, 2012; Velho *et al*, 2012). Because of the lack of effective therapies for this disease, prognosis after the development of metastases is poor (Spagnolo *et al*, 2012; Velho *et al*, 2012). For example, the median OS for patients with metastatic uveal melanoma may be as little as 3.6 months, and 5-year OS as low as 1% has been reported (Patel *et al*, 2011). In contrast, the median OS for the 23 patients with metastatic uveal melanoma enroled in our study was 12.6 months and the median PFS was 4.8 months, thereby suggesting potential for clinical activity with cabozantinib in this melanoma subtype.

The most frequent AEs in this cohort (e.g., fatigue, diarrhoea, nausea, and decreased appetite) were mainly mild to moderate in severity and were consistent with those observed in the other cohorts in the RDT. A similar safety profile is observed with other VEGFR inhibitors, and AEs are generally managed with dose holds and reductions and supportive care.

In this randomised discontinuation trial of cabozantinib, clinical activity was observed in a cohort of patients with metastatic melanoma, and toxicity was manageable and consistent with other multikinase inhibitors. The data from this trial also highlight the potential benefits of cabozantinib on both soft tissue and bone lesions in patients with metastatic melanoma. Treatment with cabozantinib was associated with encouraging PFS and OS, and reduction in the size of measurable target lesions was observed in the majority of patients with uveal, cutaneous, and mucosal melanoma. Clinical activity appeared to be independent of *BRAF* mutation status. Overall, these data suggest that targeting the VEGFR, MET, and AXL pathways with cabozantinib may lead to improved outcomes in patients with metastatic melanoma. However, because this phase II randomised discontinuation trial was underpowered to draw definitive conclusions in the cohort of patients with metastatic melanoma, additional studies of cabozantinib in this patient population are needed to confirm these results.

## Figures and Tables

**Table 1:** Baseline demographic and clinical characteristics of patients

	**Entire treated population (*****N*****=77)**
**Characteristic**	***n*****(%)**
Age (years)	
Median	65
Range	30–90
Sex	
Male	42 (55)
Female	35 (45)
Melanoma subtype	
Cutaneous	48 (62)
Uveal	23 (30)
Mucosal	6 (8)
Bone metastases	17 (22)
Known *BRAF* mutation	17 (31)^a^
Prior lines of therapy^b^	
0	26 (34)
1–2	43 (56)
⩾3	8 (10)
Prior therapies of interest	
*BRAF* inhibitor	6 (8)
MEK inhibitor	3 (4)
Ipilimumab	3 (4)

Abbreviation: MEK=mitogen-activated protein kinase (MAPK)/ERK kinase.

a Based on patients with available *BRAF* mutation data (*n*=54).

b The protocol limited prior therapies to ⩽2 prior standard or investigational chemotherapy or targeted therapy regimens in the metastatic setting.

Radiotherapy and immunotherapy (such as IL-2 and immune checkpoint inhibitors) did not count towards this restriction. The number of prior therapies summarised here includes therapies that did not count towards the limit of ⩽2 prior regimens.

**Table 2:** GNAQ/GNA11 mutation status and time on study treatment in the 23 uveal melanoma patients

**Patient #**	**GNAQ mutation**	**GNA11 mutation**	**Months on study treatment**^a^
1	UNK	UNK	24.5+
2	UNK	UNK	19.9+
3	UNK	UNK	14.9+
4	UNK	UNK	14.6+
5	UNK	UNK	11.6
6	R183Q	ND	10.3
7	UNK	UNK	9.7
8	ND	Q209L	8.7
9	UNK	UNK	6.5
10	UNK	UNK	5.0
11	UNK	UNK	4.6
12	ND	UNK	4.3
13	UNK	UNK	3.5
14	ND	R183C	3.0
15	Q209P	UNK	3.0
16	Q209P	ND	2.3
17	ND	Q209L	2.1
18	Q209P	UNK	1.8
19	UNK	UNK	1.7
20	ND	Q209L	1.4
21	Q209L	ND	1.4
22	UNK	UNK	1.4
23	UNK	UNK	1.3

Abbreviations: ND=not detected; UNK=unknown.

a Months with a plus (+) indicate patients who remain on study treatment as of the data cutoff.

**Table 3:** Most frequently reported AEs during lead-in stage regardless of causality

	**All grades (*****N*****=77)**	**Grade ⩾3**^a^ (***N*****=77)**
AE^b^	***n*** **(%)**	***n*** **(%)**
Fatigue	46 (60)	11 (14)
Diarrhoea	44 (57)	2 (3)
Nausea	39 (51)	1 (1)
Decreased appetite	35 (45)	0 (0)
Abdominal pain	24 (31)	6 (8)
Vomiting	23 (30)	2 (3)
Hypertension	22 (29)	8 (10)
Constipation	20 (26)	3 (4)
Dysgeusia	20 (26)	0 (0)
Hand-foot syndrome	19 (25)	4 (5)
Stomatitis	19 (25)	0 (0)
Aspartate aminotransferase increased	17 (22)	2 (3)
Dry mouth	17 (22)	0 (0)
Dysphonia	17 (22)	0 (0)
Rash	16 (21)	0 (0)
Weight decreased	16 (21)	0 (0)
Dyspnoea	13 (17)	3 (4)
Mucosal inflammation	13 (17)	0 (0)
Alanine aminotransferase increased	12 (16)	2 (3)
Hypomagnesaemia	12 (16)	0 (0)
Asthenia	11 (14)	4 (5)
Abdominal pain upper	11 (14)	1 (1)
Dizziness	11 (14)	1 (1)
Oral pain	11 (14)	1 (1)
Urinary tract infection	11 (14)	0 (0)
Back pain	10 (13)	4 (5)
Hypokalaemia	10 (13)	4 (5)
Pain in extremity	10 (13)	3 (4)
Dry skin	10 (13)	0 (0)

Abbreviations: AE=adverse event; CTCAE=Common Terminology Criteria for Adverse Events; MedDRA=Medical Dictionary for Regulatory Activities.

a One related grade 5 event was reported: fatal event of acute peritonitis due to diverticular perforation. Another unrelated death from an unknown cause was also reported (Figure 1).

b MedDRA v. 15.0 Preferred Terms (converted to US spelling), CTCAE v. 3.0 grading.
