# Supplementary material for: Phase II randomised discontinuation trial of the MET/VEGF receptor inhibitor cabozantinib in metastatic melanoma
Source: Br J Cancer. 2017 Jan 19;116(4):432–40. doi: 10.1038/bjc.2016.419 (PMC5318966; doi:10.1038/bjc.2016.419)
Supplement: Supplementary Information [file bjc2016419x1.docx]

**Supplementary Material**

**Supplementary Figure S1.** Schematic of randomised discontinuation trial design. Eligible patients with cutaneous, mucosal, or uveal melanoma received cabozantinib treatment during a 12-week, open-label, lead-in stage. At week 12, patients with objective tumour response by RECIST (CR or PR) continued open-label treatment with cabozantinib. Patients with PD were discontinued. Patients with SD were randomly assigned to either continued treatment with cabozantinib or matched placebo (random assignment stage). Randomly assigned patients with PD were offered to resume cabozantinib treatment if they were receiving placebo and were observed until their subsequent progression. Abbreviations: CR, complete response; PD, progressive disease; PR, partial response; RECIST, Response Evaluation Criteria in Solid Tumours; SD, stable disease.

**Supplementary Figure S2.** Bone scan effects of cabozantinib treatment. Sequential whole-body technetium methylene diphosphonate bone scintigraphy of two patients with uveal melanoma. Baseline scans show areas of increased radiotracer uptake (arrows) indicative of extensive bone metastases. Treatment with cabozantinib resulted in partial resolution of bone scans at week 6.

**Supplementary Figure S3.** Bone marker analysis. Best change from baseline in plasma CTx, a marker of bone resorption, in the subset of patients with bone metastases and at least one postbaseline CTx assessment (week 6 and/or week 12). The plot includes two patients with a medical history of bone metastases without baseline scan data. ^a^ Change from baseline exceeded 100%. Abbreviation: CTx, cross-linked C-terminal telopeptide of type 1 collagen.
