# Supplementary figures and images for: Phase II randomised discontinuation trial of the MET/VEGF receptor inhibitor cabozantinib in metastatic melanoma
Source: Br J Cancer. 2017 Jan 19;116(4):432–40. doi: 10.1038/bjc.2016.419 (PMC5318966; doi:10.1038/bjc.2016.419)

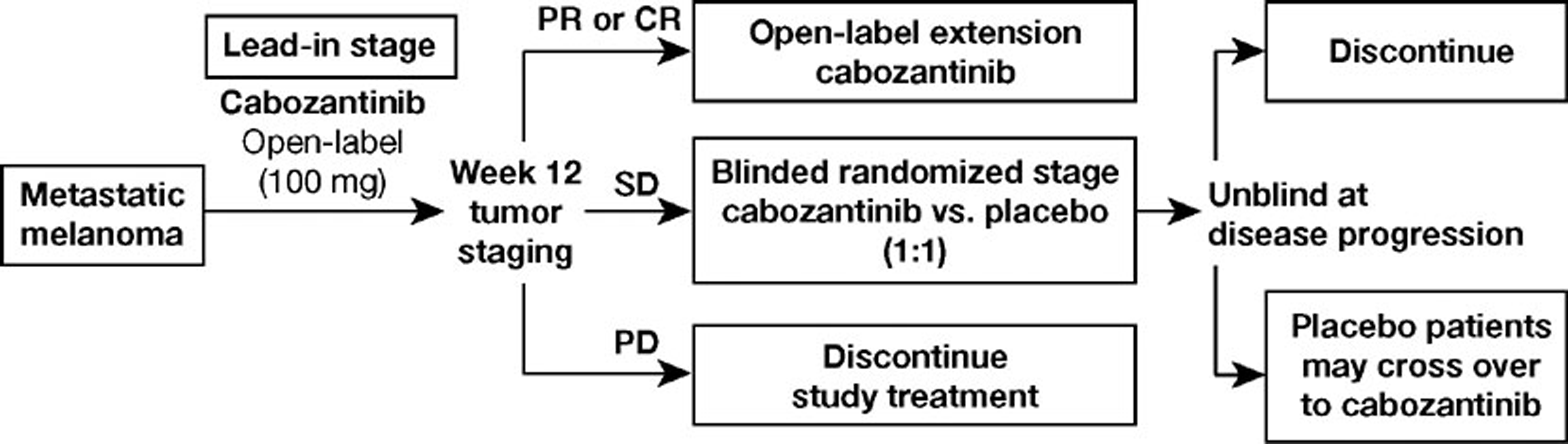

Supplement: Supplementary Information [file bjc2016419x2.tif]

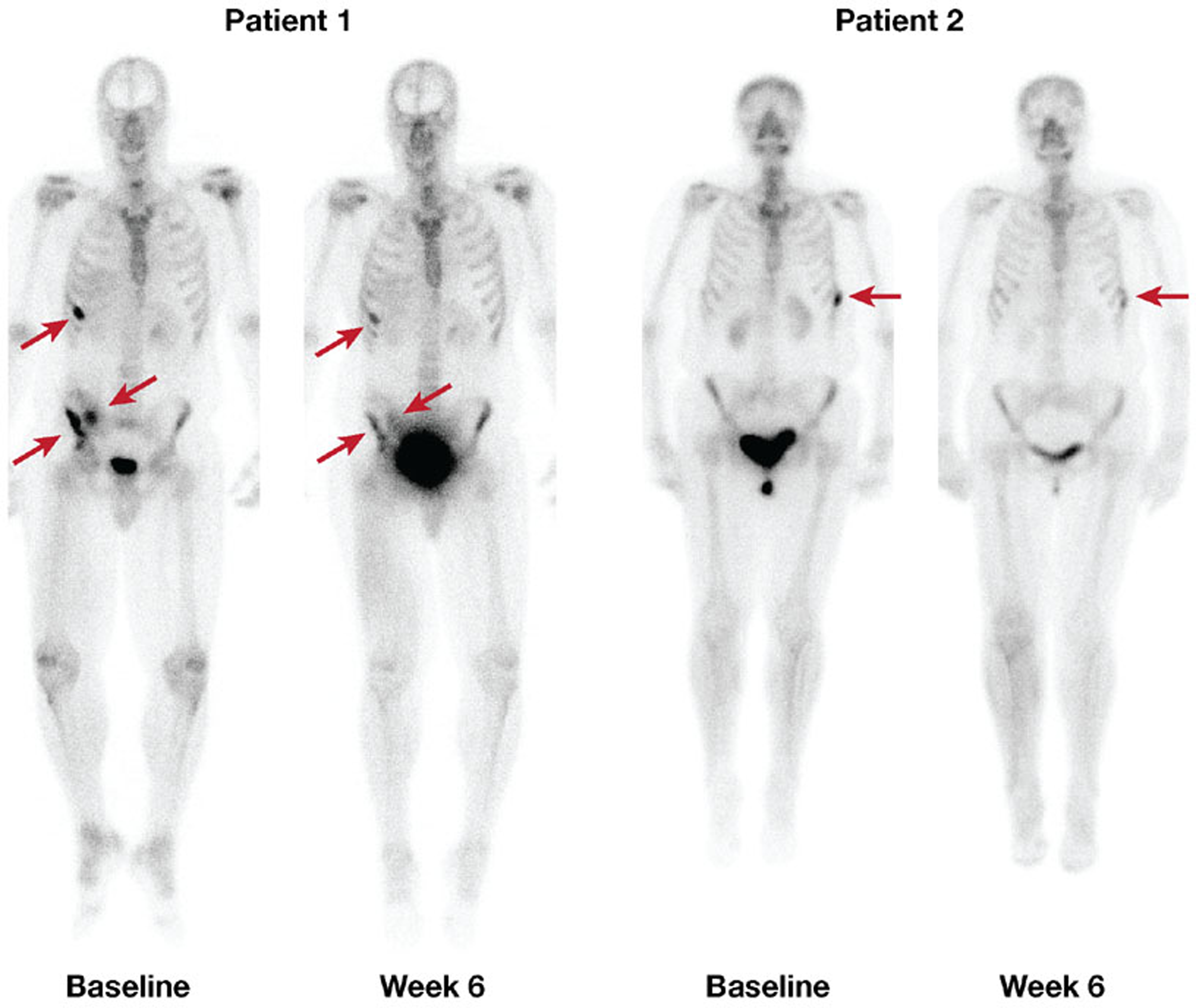

Supplement: Supplementary Information [file bjc2016419x3.tif]

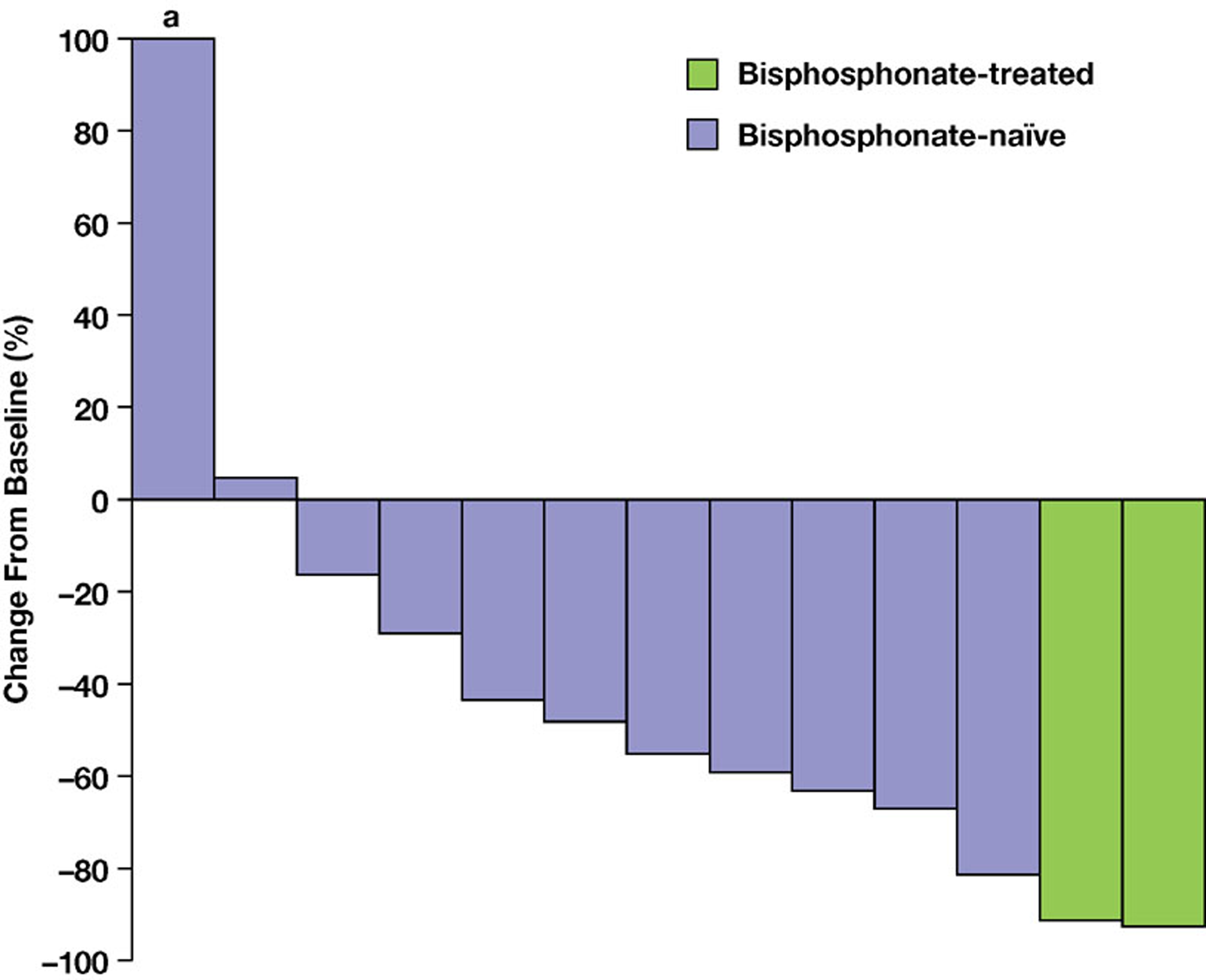

Supplement: Supplementary Information [file bjc2016419x4.tif]
